# PREVIEW: Prevention of Diabetes through Lifestyle Intervention and Population Studies in Europe and around the World. Design, Methods, and Baseline Participant Description of an Adult Cohort Enrolled into a Three-Year Randomised Clinical Trial

**DOI:** 10.3390/nu9060632

**Published:** 2017-06-20

**Authors:** Mikael Fogelholm, Thomas Meinert Larsen, Margriet Westerterp-Plantenga, Ian Macdonald, J. Alfredo Martinez, Nadka Boyadjieva, Sally Poppitt, Wolfgang Schlicht, Gareth Stratton, Jouko Sundvall, Tony Lam, Elli Jalo, Pia Christensen, Mathijs Drummen, Elizabeth Simpson, Santiago Navas-Carretero, Teodora Handjieva-Darlenska, Roslyn Muirhead, Marta P. Silvestre, Daniela Kahlert, Laura Pastor-Sanz, Jennie Brand-Miller, Anne Raben

**Affiliations:** 1 Department of Food and Environmental Sciences, University of Helsinki, 00014 Helsinki, Finland; elli.jalo@helsinki.fi; 2 Department of Nutrition, Exercise and Sports, Faculty of Science, University of Copenhagen, Rolighedsvej 30, Frederiksberg C, DK-1958 Copenhagen, Denmark; tml@nexs.ku.dk (T.M.L.); piach@nexs.ku.dk (P.C.); laura.pastor@adm.ku.dk (L.P.-S.); ara@nexs.ku.dk (A.R.); 3 Department of Human Biology, Maastricht University, P.O. Box 616, 6200 MD Maastricht, The Netherlands; m.westerterp@maastrichtuniversity.nl (M.W.-P.); m.drummen@maastrichtuniversity.nl (M.D.); 4 School of Life Sciences, Faculty of Medicine and Health Sciences, University of Nottingham, Nottingham NG7 2UH, UK; Ian.Macdonald@nottingham.ac.uk (I.M.); liz.simpson@nottingham.ac.uk (E.S.); 5 Center for Nutrition Research, University of Navarra, 31008 Pamplona, Spain; jalfmtz@unav.es (J.A.M.); snavas@unav.es (S.N.-C.); 6 CIBERobn, Instituto de Salud Carlos III, 28029 Madrid, Spain; 7 Department of pharmacology and toxicology, Medical University of Sofia, 1431 Sofia, Bulgaria; nadkaboyadjieva@gmail.com (N.B.); teodorah@abv.bg (T.H.-D.); 8 Human Nutrition Unit, School of Biological Sciences, University of Auckland, Auckland 1024, New Zealand; s.poppitt@auckland.ac.nz (S.P.); m.silvestre@auckland.ac.nz (M.P.S.); 9 Department of Exercise and Health Sciences, University of Stuttgart, 70569 Stuttgart, Germany; wolfgang.schlicht@inspo.uni-stuttgart.de; 10 School of Sport and Exercise Sciences, A.STEM Research Centre, Swansea University, Swansea SA1 8EN, UK; g.stratton@swansea.ac.uk; 11 National Institute for Health and Welfare THL, 00300 Helsinki, Finland; jouko.sundvall@thl.fi; 12 NetUnion sarl, Ave des Figuires 20, 1007 Lausanne, Switzerland; lam@netunion.com; 13 Charles Perkins Centre, University of Sydney, Sydney 2006, Australia; roslyn.muirhead@sydney.edu.au (R.M.); jennie.brandmiller@sydney.edu.au (J.B.-M.); 14 Department of Health Science, University of Education Schwäbisch Gmünd, 73525 Gmünd, Germany; daniela.kahlert@ph-gmuend.de

**Keywords:** diet, protein, carbohydrate, glycaemic index, physical activity, obesity

## Abstract

Type-2 diabetes (T2D) is one of the fastest growing chronic diseases worldwide. The PREVIEW project has been initiated to find the most effective lifestyle (diet and physical activity) for the prevention of T2D, in overweight and obese participants with increased risk for T2D. The study is a three-year multi-centre, 2 × 2 factorial, randomised controlled trial. The impact of a high-protein, low-glycaemic index (GI) vs. moderate protein, moderate-GI diet in combination with moderate or high-intensity physical activity on the incidence of T2D and the related clinical end-points are investigated. The intervention started with a two-month weight reduction using a low-calorie diet, followed by a randomised 34-month weight maintenance phase comprising four treatment arms. Eight intervention centres are participating (Denmark, Finland, United Kingdom, The Netherlands, Spain, Bulgaria, Australia, and New Zealand). Data from blood specimens, urine, faeces, questionnaires, diaries, body composition assessments, and accelerometers are collected at months 0, 2, 6, 12, 18, 24, and 36. In total, 2326 adults were recruited. The mean age was 51.6 (SD 11.6) years, 67% were women. PREVIEW is, to date, the largest multinational trial to address the prevention of T2D in pre-diabetic adults through diet and exercise intervention. Participants will complete the final intervention in March, 2018.

## 1. Introduction

Type-2 diabetes (T2D) is a disease associated with serious comorbidities, including microvascular (retinopathy, nephropathy, neuropathy) and macrovascular (cardiovascular) events [1]. The estimated global prevalence is approximately 8% and a prediction suggests that this will increase by 55% up to the year 2035 [2,3]. An important risk factor for T2D is obesity (BMI > 30 kg/m^2^) predicting a more than 10-fold increase in incidence compared to normal weight [4]. Weight gain during adulthood is also an independent risk factor for T2D [5], as are genetic inheritance, unhealthy dietary habits, and insufficient physical activity [6,7,8].

Long-term studies have shown benefits of a lifestyle intervention (diet and exercise), on T2D incidence in China [9], USA [10], and Finland [11]. Lifestyle change (diet, physical activity, weight loss) may reduce the incidence of T2D by 28–59% [12]. The American Diabetes Prevention Program (DPP) [13], the Finnish Diabetes Prevention Study (DPS) [14], and the Chinese Da Qing Diabetes Prevention Study [15] were all designed to produce weight loss by prescribing a higher carbohydrate (CHO) (>50 percent of energy (E%) from CHO), low-fat (<30 E%) diet approach, which reflected the understanding of a prudent diet 20 years ago. No attention was paid to glycaemic index (GI), per se, and, to date, no studies have investigated the role of GI for prevention of type-2 diabetes.

Other dietary prescriptions that produce significant and sustainable weight loss may also be effective in T2D prevention. Current international recommendations include lower ranges for CHO intake [16] and a recommendation to choose lower GI foods [17]. A combination of lower CHO (45 E%), higher protein, together with lower GI, might be the optimal diet for prevention of T2D [18], perhaps related to sustained weight loss as shown in medium term trials [19]. To date these hypotheses have not been tested in large trials of long duration [20].

The program for physical activity in the trials described above followed the international public health recommendations, that is, a total of approximately 150 min per week of moderate-intensity aerobic activities or 75 min of vigorous intensity activity [10,14]. A question, not examined in earlier studies, is whether the metabolic responses are different between higher- and lower-intensity exercise programs. Moderate-intensity exercise relies relatively more on fat oxidation, whereas vigorous-intensity exercise relies more on CHO oxidation and use of intramuscular substrates [21]. Houmard et al. found that total exercise time, not intensity or exercise energy expenditure, was associated with the greatest improvement of insulin sensitivity in obese participants [22]. However, the hypothesis that physical activity with different intensity levels may differentially affect T2D prevention has yet to be tested in any large-scale intervention.

Since obesity is a strong risk factor for T2D, any successful prevention program should be able to prevent weight regain in individuals after a significant weight loss. The high heterogeneity of dietary intervention design prevents firm conclusions being drawn regarding preferred macronutrient composition [23]. Notably a recent multi-centre trial ‘DiOGenes’ (Diet, Obesity, and Genes) identified a higher-protein, moderate-CHO, and low-GI diet as superior to other diets of varying macronutrient composition in preventing weight regain over six months [19] and in a smaller subset over 12 months [24], after two months of rapid weight loss.

Despite the evidence that a lifestyle program combining prudent diet, increased physical activity and weight loss reduces the risk for T2D in susceptible individuals [12], important details remain unanswered. These include the long-term effects and sustainability of diets higher in protein with a lower glycaemic load, combined with the effects of higher intensity exercise. The present paper describes PREVention of diabetes through lifestyle Intervention and population studies in Europe and around the World (PREVIEW), a large multi-centre international randomised controlled trial in adults designed to answer these questions.

## 2. Methods

### 2.1. Aims of the Study

The aim is to determine the effects and interactions of two diets and two physical activity programmes on the prevention of T2D in overweight, pre-diabetic adults, who have undergone a short period of significant weight loss. Our primary hypothesis is that a higher protein, lower CHO/low GI diet (based on the DiOGenes study [19]) will be superior in preventing T2D when compared with a moderate protein, higher CHO/moderate GI diet (based on the DPS and DPP studies [10,14]). We also hypothesise that high-intensity physical activity will be superior compared to moderate-intensity physical activity [25].

Each participant receives one of the two dietary programs, and one of the two physical activity programs, thus, we have four groups (high protein diet and high-intensity physical activity; moderate protein diet and high-intensity physical activity; high protein diet and moderate-intensity physical activity; moderate-protein diet and moderate-intensity physical activity). The majority of outcomes will be analysed by using these four arms. The primary endpoint and statistical power calculations are based on a two-arm design (diets compared against each other).

### 2.2. Primary and Secondary Endpoints

The primary endpoint is incidence of T2D in high vs. moderate protein diet measured over a 36-month intervention period, based on the WHO criteria [26] of either (i) oral glucose tolerance test (OGTT) with fasting plasma glucose (FPG) > 7.0 mmol/L and/or 2-h post prandial (75 g glucose load) plasma glucose ≥11.1 mmol/L; or (ii) T2D diagnosed by a medical doctor between the clinical investigation days (CID) of PREVIEW, by using random plasma glucose ≥11.1 mmol/L in the presence of symptoms of diabetes, OGTT, or glycated haemoglobin (HbA1c). Asymptomatic individuals with a single abnormal value will have to repeat the test within 2–4 weeks to confirm the T2D diagnosis. The secondary endpoints include changes in HbA1c, body weight, body mass index (BMI), waist, and thigh circumference, body composition, insulin sensitivity, including Matsuda Index [27], glucose tolerance assessed by the area under the curve during OGTT, blood pressure, serum lipids, C-reactive protein, liver enzymes, perceived quality of life and work ability, habitual well-being, sleep, chronic stress, and subjective appetite sensations.

Other endpoints assessed by sub-group studies include liver fat content using magnetic resonance imaging (MRI) and proton magnetic resonance spectroscopy (H-MRS); colorectal cancer risk assessed from faecal markers; gut microbiome community assessment from faecal collections; maximal oxygen uptake capacity (VO_2_ max); urine metabolite profiles using metabolomic techniques and food reward outcomes.

### 2.3. Study Setting and Design

The PREVIEW intervention study for adult participants has eight study sites: University of Copenhagen (Denmark), University of Helsinki (Finland), University of Maastricht (The Netherlands), University of Nottingham (UK), University of Navarra (Spain), Medical University of Sofia (Bulgaria), University of Sydney (Australia), and University of Auckland (New Zealand).

**Figure 1:**
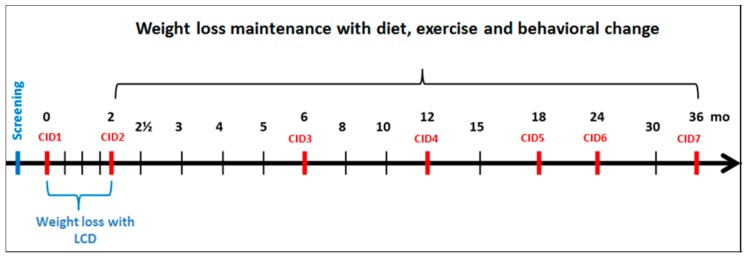
PREVIEW intervention: the general study design.

The 36-month intervention consists of two phases (Figure 1): a two-month period of rapid weight reduction achieved using a commercial low-calorie diet (about 800 kcal/day), followed by a 34-month randomised lifestyle (diet and physical activity) intervention phase for weight loss maintenance.

Clinical investigation days (CID) are conducted throughout the intervention, from CID1 (baseline) to CID7 (end of trial). At CID visits, anthropometry, blood tests, and questionnaires are performed and collection of completed diet records, accelerometers, and 24-h urine samples is done. Adverse (AE) and serious adverse events (SAE) and concomitant medications are recorded. In addition, a total of 17 group visits, leaded by instructors, are held throughout the trial to support lifestyle modification.

The CID assessments and group visits are conducted within University settings or associated Clinics. Participants follow the diet and physical activity counselling advice in a “real-life” setting without daily supervision from researchers.

### 2.4. Participants, Recruitment, and Randomisation

The inclusion criteria were: age 25–70 years (from mid-2013 to mid-2014 individuals aged 25–45 and 55–70 years were enrolled, and from mid-2014 onwards additionally age-group 45–54 years); BMI > 25 kg/m^2^; pre-diabetes confirmed by an OGTT using the American Diabetes Association (ADA) criteria (13): (i) increased fasting glucose (IFG), with venous plasma glucose concentration of 5.6–6.9 mmol/L when fasted; and/or (ii) impaired glucose tolerance (IGT), with venous plasma glucose concentration of 7.8–11.0 mmol/L at 2 h after oral administration of standard 75 g glucose dose, and fasting plasma glucose <7.0 mmol/L. The main exclusion criteria were T2D, and any illness and/or medication with known or potential effect on compliance (e.g., unable to follow the physical activity program) or the main outcomes. A complete list of inclusion and exclusion criteria is presented as Supplementary Table S1.

Participants were recruited using multiple methods across the eight study sites, e.g., newspaper advertisements, newsletters, radio and television advertisements/interviews, and direct contact with primary and occupational health care providers. Interested individuals were contacted for the pre-screening. In the interview, inclusion and exclusion criteria were queried, including the Finnish Diabetes Risk Score [28] assessment. Potential participants were given written and oral information. Signed informed consent was required prior to commencement of laboratory screening.

The laboratory screening comprised measurements of weight, height, resting blood pressure, electrocardiography (in those aged 55 years or more), and an OGTT. A fasting blood sample was collected from the ante-cubital vein for later assessment of full inclusion and exclusion criteria, whilst glucose concentration was immediately analysed at each study site (HemoCue™, Angelholm, Sweden; Reflotron™, Roche diagnostics, Switzerland; or EML105 Radiometer, Copenhagen). Participants were then given a standard glucose drink (75 g glucose, dissolved in 300 mL water), which they had to take within 3–5 min, and a second venous blood sample was collected after 2 h. No other food or drinks or smoking were allowed and participants were required to remain sedentary during the test. The 0 and 2 h glucose concentration were used to identify those with pre-diabetes. Potentially eligible participants had fasting blood samples analysed to assess safety with haemoglobin, creatinine and alanine (ALT)/aspartate transaminase (AST).

Upon confirmation of eligibility, participants were enrolled into the trial and randomised to one of the four treatment groups. Randomisation was stratified by gender and age group (25–45, 46–54, and 55–70 years of age), and sequentially assigned from each stratum to different interventions, hence, securing an even distribution of gender and age group over the four intervention arms in each centre.

### 2.5. Description of Interventions

#### 2.5.1. Low-Calorie Diet (LCD)

The trial started with a two-month (eight-week) weight reduction program using a commercial LCD, with a requirement to lose >8% initial body weight in order to continue to the weight maintenance phase. The LCD consisted of 3.4 MJ (800 kcal), 15–20 E% fat, 35–40 E% protein (84 g protein), and 45–50 E% CHO. The daily diet comprised of 4 × 40 g Cambridge Weight Plan^®^ meal replacement sachets (Cambridge Weight Plan Ltd., Corby, UK), three of which were dissolved in 250 mL low fat milk ,or similar lactose-free alternatives, and one in 250 mL water. Energy-free drinks were permitted. Moreover, a maximum of 400 g of non-starchy, low-CHO vegetables, such as lettuce, asparagus, broccoli, celery, cucumber, mushrooms, radish, tomato, and watercress could be consumed.

During the LCD, participants attended group visits at weeks 2, 4, 6, and 8. Body weight, AE, SAE, and concomitant medications were recorded, LCD sachets dispensed, and dietary and behavioural instructions given. No specific instructions on physical activity were given during the LCD weight-reduction phase. Upon completion of the two months (CID2), participants who failed to reach the target weight reduction (i.e., >8% of initial body weight) were excluded from the intervention.

#### 2.5.2. Weight Maintenance Phase: Intervention Diets

The two intervention diets are described in Table 1. The moderate protein (MP) diet is based on the DPS-dietary advice [14] aiming to reach a moderate protein (15 E%) and higher CHO (55 E%) macronutrient distribution with at least moderate dietary GI (>56), following current recommendations for prevention of T2D [17]. The (HP) diet has a higher protein (25 E%) and moderate CHO (45 E%) distribution with lower dietary GI (<50), based on the most successful weight-loss maintenance diet in the DiOGenes study [19]. Protein intake is higher and CHO intake is lower than the recommended range for prevention of T2D [16,17].

Both intervention diets are moderate in fat (30 E%) and the target macronutrient profile and food choices are supported by evidence for prevention of weight gain and/or T2D [8,23]. Notably, increased intake of sugar-rich foods or refined grains is not encouraged as a means to reach the higher CHO level, nor is increased consumption of red meat encouraged within the higher protein diet.

The diets are consumed ad libitum with respect to energy, with no provision of an individual target for daily energy intake. Self-monitoring of total energy consumption is not required. However, participants are instructed about controlling portion sizes of specific food types in order to achieve the macronutrient and GI prescriptions, and in self-monitoring and adjustment of portion sizes in general, in order to maintain their body weight loss. They are also encouraged to follow a regular meal pattern. Additional weight reduction is allowed, but without anything other than adherence to the maintenance diet and physical activity regimens.

The participants are given examples of daily eating plans with foods in appropriate proportions to reflect the macronutrient and GI requirements of the two interventions. A food-exchange list assists in self-selected variety, whilst preserving the required macronutrient and GI levels. Cooking books (one for each diet) with recipes suitable for all countries were specifically prepared for PREVIEW.

#### 2.5.3. Weight Maintenance Phase: Physical Activity Programmes

The trial has two physical activity interventions with a similar target for energy expenditure (>4.2 MJ/week, >1000 kcal/week), comprising high-intensity (HI) exercise or moderate-intensity (MI) exercise, as shown in Table 2. Measured heart rate using a heart rate monitor or wrist palpation, and/or perceived exertion using the Borg scale [29], are the principal methods of controlling the intensity. The participants may choose from several exercise options with similar level of metabolic turnover (energy expenditure divided by resting metabolic rate, i.e., MET values). The specific advice is based on the U.S. Centres for Disease Control and Prevention (CDC) recommendations of 75 min high-intensity (HI) or 150 min moderate-intensity (MI) physical activity weekly [30]. We developed a leaflet and other written instruction materials for the two PA groups. Physical activity is generally not supervised by the PREVIEW team, but participants are allowed to join supervised exercise groups of their own choice.

A critical issue in PREVIEW is that many participants may be morbidly obese (BMI > 40) and, therefore, their ability to cope with a high-intensity exercise program is likely to be limited, and even risky. We addressed this point during the recruitments by specifically asking about perceived competence in coping with our program, and by ECG in all volunteers aged >55 years. Moreover, significant weight reduction (>8% of baseline body weight) during the first two months’ LCD period will also simultaneously decrease the cardiovascular risks. The flexibility of our exercise program (only target energy expenditure is specified, the modes of exercise are due to the participant) is also likely to improve safety and adherence.

#### 2.5.4. Group Visits and the Behavioural Modification Program

Group visits (8–12 individuals), are conducted throughout the three year intervention to deliver the behaviour modification information in relation to diet and physical activity [31]. There are 17 group visits, each 1–2 h, with decreasing frequency as the trial progresses. The behaviour modification programme is developed based on theories and evidence from health psychology and behaviour change [32,33,34]. For example, participants’ beliefs about the consequences of behaviour (i.e., outcome expectancies), their intention to change their behaviour in the long run, and their belief in their ability to achieve the behaviour change goals (self-efficacy) are relevant predictors of successful behaviour change. Counsellors may apply respective behaviour change techniques [35] that are scheduled to common stages of behaviour change [36].

At the beginning of the weight-maintenance phase (i.e., month 2), the participants are instructed on how to plan, to start, and to follow the physical activity programme. In the group sessions, the participants are also instructed on basic principles of increasing physical activity and in motivational and self-regulative behaviour techniques to overcome barriers to exercise and behaviour modification. Stretching and home-based muscle-conditioning exercises are also supervised in a group-based session accompanied with written educational material [31].

### 2.6. Collection of Data and Description of Analyses

Data are collected from biological specimens (blood, urine, faecal), self-administered records and questionnaires, and an activity-monitoring device (ActiGraph GT3X accelerometer; ActiGraph, Pensacola, FL, USA) (see Table 3 with a description of timing). The CIDs are scheduled for a specific week and the aim is to make the measurements as precisely as scheduled. To accommodate as complete a data collection as possible we allow the following visit windows: month 2: −3 to +5 days; month 6: ±1 weeks; month 12: ±2 weeks; the remaining measurement points: ±4 weeks.

Blood samples are initially stored locally at −80 °C, then transported and analysed centrally at the National Institution for Health and Welfare (THL) in Helsinki, Finland. Diet records are analysed at each site using local food composition data and software. If available, local GI data for individual food items are used, and when not available, generic global GI data are used. Accelerometer data are downloaded at local sites, and collated and analysed centrally at the Swansea University, Wales, UK.

All questionnaires used in PREVIEW were prepared in English, then translated into the local language in Finland, Denmark, The Netherlands, Spain, and Bulgaria using authorized translators. A second authorized translator then back-translated the local versions to English, with this iterative process repeated until a final version of sufficient quality was obtained.

### 2.7. Data Management

All data are stored in a central project database at the University of Copenhagen. The central database ensures standardized handling and storing of data and the possibility for easy extraction and delivery of data both within and after the official project period (2013–2018).

Currently, the database receives input from four data sources on a regular basis: (1) All immediate data measured (e.g., anthropometrics, blood glucose) and interviewed (e.g., use of medication) during the CIDs and entered into OpenClinica server (electronic case report form); (2) data on social-cognitive determinants of behaviour, on cultural and socio-demographics, as well as socio-economic components, are collected by the questionnaire delivery platform (QDP), designed for PREVIEW by NetUnion. The participants enter their own data into the QDP. A paper version of the questionnaires is also available; (3) physical activity is reported using the Baecke inventory, and an electronic physical activity log (PAL), designed by Swansea University, University of Stuttgart, and implemented by NetUnion; (4) the Central Lab at the National Institute for Health and Welfare (THL) enters all laboratory analyses into the data hub. Data from analyses of the ActiGraph data accelerometers, from food diaries, and from the maximal oxygen uptake (VO_2_ max) analyses are imported from all sites.

### 2.8. Governance and Quality Management

The intervention trial is led by Prof. Fogelholm at the University of Helsinki, in collaboration with the project coordinator, Prof. Raben at the University of Copenhagen. In this large, international multi-centre trial, we are collaborating intensively to ensure data collection of high-quality and consistency of the intervention across all sites.

Specific working groups were formed with relevant site representatives. The purpose of these working groups is to discuss and agree on questions related to dietary topics, physical activity, data management, and other methodological and medical issues.

During the recruitment phase, principal investigators from each centre participated in a monthly teleconference, which continues at regular intervals throughout the intervention.

The core personnel for each site meet annually at a three-day general assembly for the full PREVIEW consortium. PREVIEW has a website [37] with both public access and a restricted area for the PREVIEW researchers.

An electronic trial master file with relevant documents has been designed and is maintained by the University of Copenhagen within the private part of the PREVIEW website. All written study material is uploaded and made available at the PREVIEW website private area, including the protocol and amendments, standard operating procedures (SOPs), and instruction materials for the intervention subjects, in order ensure that comparable methods are followed across individual sites. The SOPs are reviewed and revised as needed and also new SOPs are prepared, if necessary.

Representatives from each intervention site participated in two training sessions, each of 2–3 days duration, in 2013. One session focussed on the main study protocol, the CID protocols, and all outcome measurements (University of Copenhagen). The other session focused on instructor training in group counselling (behaviour change) methods (University of Stuttgart). Attendees then trained their local staff.

### 2.9. Statistical Power and Basic Analyses

The anticipated three-year incidence of T2D in the PREVIEW trial is 21%, based on data from the Finnish DPS and US DPP [10,11]. The power calculation was derived for comparison of the two dietary interventions (HP vs. MP).

It was hypothesized that a risk reduction of one quarter (1/4) in the MP group would reduce the incidence of T2D incidence from 21% to 16%, and that a risk reduction of one half (1/2) in the HP group would reduce the incidence of T2D from 21% to 10.5%. Consequently, the sample size required to detect this difference in T2D incidence (16% vs. 10.5%) was at least 649 per diet group or 1298 participants in total (for a two-sided comparison with a power (1-ß) of 80% and *p* < 0.05), with a 10% drop-out during the first 10 months from month 2 (CID2) onwards, and another 20% drop-out between months 12 (CID4) and 36 (CID7). Thus, the number of participants needed for the intervention was 1802. To allow an estimated drop-out of 25% as a result of failure to lose >8% of initial body weight during the two-month LCD period, the number of participants required to be enrolled into PREVIEW was initially estimated to be 2403.

The primary data are analysed statistically using the principle of ‘intention-to-treat’ (ITT cohort) and also as a completers’ cohort. A ‘completer’ is defined as a participant who has remained in the trial for the full three year intervention period, or who has been diagnosed with T2D before the end of the intervention.

The primary outcome in the adults’ trial is incidence of T2D. For statistical analysis assessing the effect of the two diets on the T2D is a ‘semi-parametric Cox proportional hazards regression model’. Missing data are addressed using hot-deck imputation. Missing covariate information is addressed using multiple imputation. Sensitivity analyses (e.g., complete-case analyses without drop-outs) will be carried out to assess if censoring was informative or non-informative.

For statistical analysis of the continuous secondary outcomes (e.g., blood chemistry, anthropometrics, etc.) a ‘linear mixed model’ is used. For the categorical outcomes (e.g., sex, educational attainment, proportion of subjects maintaining a defined weight loss, etc.), the type of statistical analysis is ‘logistic’ or ‘ordinal mixed-effects model’. The parameter of interest is the difference in odds ratio between the intervention groups. Although the main statistical analyses will be done by using the entire cohort, one of the most important stratified analyses will use an age-group (e.g., above and below 65 years) stratification. By comparing older against younger participants we might obtain new insight on whether dietary protein content in this respect has different effects on, e.g., body composition, weight, and clinical variables.

### 2.10. Ethical Issues

The study protocol and amendments were reviewed and approved by local Human Ethics Committees at all study sites. The work of PREVIEW is carried out in full compliance with the relevant requirements of the latest version of the Declaration of Helsinki (59th WMA General Assembly, Seoul, Korea, October 2008), and the ICH-GCP, The International Conference on Harmonisation (ICH) for Good Clinical Practice to the extent that this is possible and relevant. All participants provided written informed consent prior to commencing screening procedures in clinic. All information obtained during the trial is handled according to local regulations and the European Directive 95/46/CE (directive on protection of individuals with regard to the processing of personal data and on the free movement of such data). The trial is registered with ClinicalTrials.gov, NCT01777893.

## 3. Results

**Figure 2:**
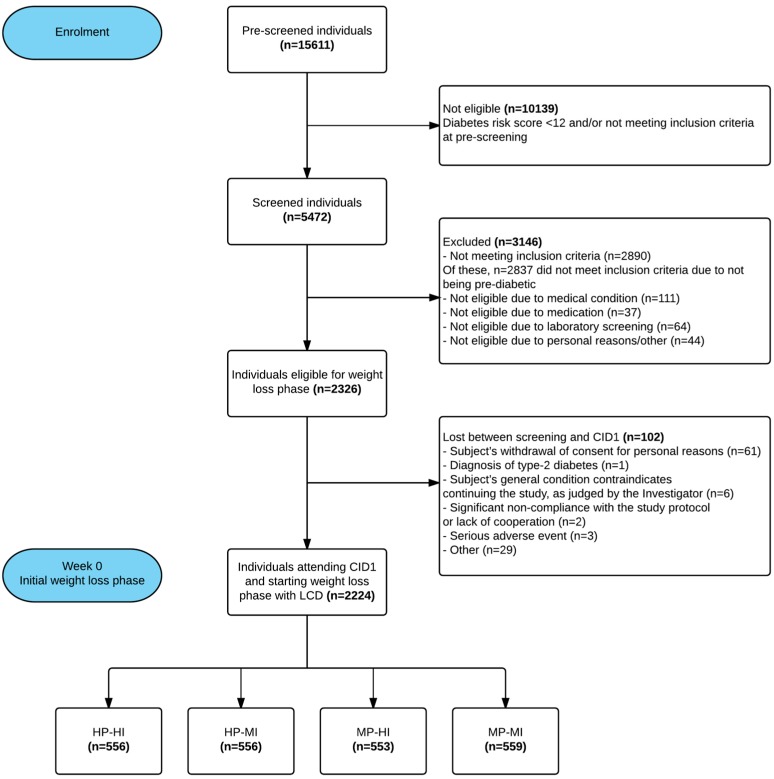
PREVIEW intervention: the subjects’ flowchart.

As PREVIEW is an on-going trial, only results obtained from participant screening and baseline phases are presented here. Screening was conducted from June 2013 to February 2015. On average, 35% of the pre-screened individuals were eligible for the laboratory screening. Further, 43% of the screened participants were found to be eligible for the trial. In total, 2326 overweight, pre-diabetic adults were enrolled and randomised into the trial. This was 97% of the original pre-specified target (Figure 2). Approximately half of the participants were 55–70 years at baseline (Table 4).

Baseline characteristics from blood biochemistry and anthropometric assessments are shown assigned to each intervention group in Table 5. The basic characteristics of the groups are similar. A notable feature of the participants is that the mean baseline fasting glucose concentration was approximately at the mid of the eligibility range, whereas the mean 2-h glucose concentration was at the lower cut-off point. According to the OGTT laboratory data, 1389 (62%) of all participants had increased fasting glucose at baseline, 506 (23%) had impaired glucose tolerance and 286 (13%) had both of these pre-diabetic indicators. At baseline (CID1), 25 participants (1%)—who all had been diagnosed with pre-diabetes at screening—were not diagnosed with pre-diabetes anymore. The prevalence of pre-diabetes described above were not significantly different between the four study groups.

## 4. Discussion

To our knowledge, PREVIEW is the first trial of its kind comparing two potentially effective interventions, a novel higher protein/low GI diet vs. current best practice moderate protein, higher CHO/moderate GI diet, in order to determine whether there is a more efficient lifestyle strategy to prevent T2D. Moreover, previous studies have neither used an effective weight-loss phase by LCD as a start of the intervention, nor a multi-country design.

Our inclusion criteria for “pre-diabetes” differed from the Finnish DPS. Here, IGT was an unconditional requirement without limits for IFG [14]. In the US DPP, both IGT and IFG were required [38] and the lower limit for IFG was 5.3 mmol/L (vs. 5.6 mmol/L in PREVIEW). It is unclear if the differences in diagnostic criteria between these studies have any major effects on the outcome. In addition to the diagnostic cut-offs, per se, and the distribution of results within the diagnostic criteria (i.e., above the lower and below the upper cut-off points) may have an effect on the outcome [39]. In PREVIEW, a majority of the subjects were eligible due to higher fasting blood glucose, rather than impaired glucose tolerance (higher 2-h value). A small proportion (1%) were no longer diagnosed with pre-diabetes at baseline. This may be explained by change of method (HemoCue™ or Reflotron™ at screening, or the laboratory assessment at baseline), to normal day-to-day variance in the assessed variables, or to a change in lifestyle after being accepted as a participant to PREVIEW. For future studies of T2D prevention, a single measurement of HbA1c, which is becoming the standard clinical practice in many countries may save both time and costs [40]. Still, there remains some controversy as to the utility of HbA1c when compared with standard OGTT as a diagnostic tool [41].

PREVIEW is a much larger trial than both the Finnish DPS (*n* = 522) [11] and Chinese Da Qing study (*n* = 577) [9], although smaller than the DPP (*n* = 3234) [10]. However, in the DPP a third of the participants received ‘Metformin’ in addition to dietary advice; hence, the number of participants without medical treatment, but adhering to lifestyle intervention (diet and physical activity) was 2161, which is similar to PREVIEW. Of the above interventions, the geographical and ethnic variation is greatest in PREVIEW. The age-range of participants in DPP and DPS was 33–67 years, slightly narrower than in PREVIEW, but the mean age of participants is similar across the studies (50–55 years). The large proportion of older participants in PREVIEW, (>55 years), was expected since the risk for T2D increases with age [2], and due to the growing health consciousness of this age group, many of whom are retired and have the time to participate in a demanding intervention. The smaller proportion of middle age-adults is also explained by later initiation of recruitment in this age group, compared with younger and older participants. In general, finding an adequate number of pre-diabetic subjects was a real challenge in most countries, given the “hidden” status of this condition. Thus, recruitment took about three times as long as planned from the beginning of the project.

One major challenge in PREVIEW is related to adherence to the diet and physical activity programs. The HP-diet is novel and, with the higher protein content, also somewhat outside the current boundaries of nutritional guidelines [42,43]. Whether the HP diet results in better adherence than the MP diet with high CHO and whole-grain cereal intakes is one of the key interests in PREVIEW. Nutrient intakes and food consumption patterns will be assessed in PREVIEW by repeated 4-d diet-records. The poor accuracy of dietary assessment is well known [44], but it is expected that the difference in dietary protein and CHO intakes should be sufficiently large to be detected using this method. Moreover, protein intake is verified using 24 h urinary nitrogen excretion [45].

It may be more difficult to create a verifiable difference for GI than for protein-to-CHO ratio. Whilst GI values of foods have been shown to provide a good summary of postprandial glycaemia [46], difficulties in attributing GI values to foods for which there are no validated data available may add to the variability [47], particularly in a multi-centre intervention such as PREVIEW. In DiOGenes, the reported observed difference in mean dietary GI was small (56 vs. 60 units) [19] which increases the requirements of precision.

The physical activity intervention in PREVIEW is not a supervised training programme. Participants are expected to integrate activity into their daily lives and use local opportunities to achieve their goals. To improve adherence, there is flexibility with the type of activities chosen. A combination of measures of physical activity is used to analyse the compliance to the type of intervention and our methods allow a description of activities that were used to achieve this.

It is probable that the HI-program will be more challenging in the long-term. Warming-up, cooling-down, muscular conditioning exercises, and stretching are carefully explained to the participants in order to decrease the risk for injuries. It should be noted that the HI-program in PREVIEW is not high-intensity interval-training (HIIT). While there are some data on potential benefits from HIIT on cardiovascular function and glucose metabolism [48], we considered the data on feasibility and long-term maintenance of this kind of training still too limited.

In addition to good compliance of the programmes, keeping the drop-out rate as low as possible will be challenging. Frequent contact with the research staff is one way to reduce the drop-out rate. However, PREVIEW has been planned to study how behavioural change is realized under ‘real-life’ conditions and, hence, the fading visit design where group visits are infrequent during years two and three of the intervention. Adherence is encouraged through a number of practices, including use of specific behavioural change techniques [31], such as implementation intentions, or Facebook groups, one for each randomised group, to promote attendance at group visits and CIDs. In addition, the sites can also conduct general information lectures, physical activity sessions, and/or send a newsletter to the participants, once or twice a year.

Compared to DPS and DPP studies, a particular feature of PREVIEW is related to the different settings in which the intervention is conducted, including not only genetic background, but also attitudes, norms, and socio-economic features. Although the participating countries are well-developed, considerable variations exist, e.g., regarding food attitudes and habits, as well as traditions of practicing physical activity.

The unique feature in the PREVIEW intervention is the direct comparison of two potentially efficacious diet and physical activity intervention programs. Hence, PREVIEW has a clear potential to identify a recommended optimal diet and physical activity programme to prevent T2D, a programme which is also suitable across different countries. It is also possible, however, that PREVIEW data will demonstrate that there are several, equally efficacious alternatives. Clearly, both answers are important from a public health viewpoint.

## Figures and Tables

**Table 1:** Description of the PREVIEW dietary interventions.

	Higher Protein (25 E% ^a^) Moderate Carbohydrate (45 E%) Low GI ^b^ (≤50) Diet	Moderate Protein (15 E%) Higher Carbohydrate (55 E%) Medium GI (≥56) Diet
Comparison between the groups	Protein intake higher Carbohydrate intake lower GI lower	Protein intake lower Carbohydrate intake higher GI medium
Food items with increased use (relative to the other group)	Whole-grain cereals with low GI Pasta Low-fat dairy products Poultry Fish Legumes	Whole-grain cereals with moderate/high GI, e.g., bread Potatoes, sweet potatoes, couscous, rice Bananas
Similar use	Most fruits and vegetables Vegetable oils, margarine Red meat (decreased in both) Sugar-sweetened beverages (decreased in both)	

^a^ E%, percentage of energy; ^b^ GI, glycaemic index.

**Table 2:** Description of the physical activity interventions.

	High-Intensity Physical Activity (HI)	Moderate-Intensity Physical Activity (MI)
Heart rate	76–90% HRmax ^a^ or 61–80% HRR ^b^	60–75% HRmax or 45–60% HRR
Examples of activities (these may vary depending on the fitness level of the participant)	Bicycling, vigorous effort Strenuous ball games Aerobics with very vigorous effort, e.g., with extra weights Jogging > 8 km/h Swimming, vigorous effort Cross-country skiing	Bicycling, moderate effort Leisurely ball games Most conditioning exercises (aerobic, power yoga, etc.) Brisk walking (4–6 km/h) Swimming, recreational Downhill skiing
Weekly duration (in total)	at least 75 min	at least 150 min
Recommended weekly frequency	2–3 times	3–5 times
Daily duration (guideline)	25–40 min	30–50 min (may be broken down into shorter sessions)
Additional exercises	Muscle conditioning exercises, by using own weight: twice weekly at home, 15–20 min per session. Stretching: twice weekly, 15–20 min per session	

^a^ HRmax = max heart rate, defined as 220—age (220 in children under 16 years of age); ^b^ HRR = heart rate reserve, defined as the difference between measured resting HR and estimated HRmax.

**Table 3:** Overview of data collection methods at different clinical investigation days (CID) in PREVIEW.

Outcome	Data Collection Method	Assessment Time-Points (Month)						
		0	2	6	12	18	24	36
		CID1	CID2	CID3	CID4	CID5	CID6	CID7
Glucose tolerance/diagnosis of T2D	75 g oral glucose tolerance test	×		×	×		×	×
Blood chemistry (lipid metabolism, glucose metabolism, inflammation markers, etc.)	Fasting venous blood specimen	×	×	×	×	×	×	×
Urinary nitrogen	24-h urine collection	×		×	×		×	×
Risk markers for colon cancer (e.g., Short Chain Fatty Acids)	3-day faecal collection ^a^	×			×			
Gut microbiota	Faecal spot sample ^a^	×			×			
Weight, height, BMI and anthropometrics	Weight; height (week 0 and 156); waist and hip circumference	×	×	×	×	×	×	×
Body composition	Body composition by DXA, BodPod or Bioelectrical impedance (BIA)	×	×	×	×		×	×
Blood pressure and resting heart rate	Resting blood pressure and heart rate	×	×	×	×	×	×	×
Nutrient intakes, dietary GI and food consumption	4-day food record	×		×	×		×	×
Physical activity	7-day accelerometer, 7-day physical activity log, Baecke questionnaire	×		×	×		×	×
Maximal oxygen uptake	VO_2_ max test by ergometer or treadmill ^b^	×		×			×	
Psycho-social mediators and moderators health behaviour	Several questionnaires (listed with references in Supplementary Table S2)	×	×	×	×		×	×
Eating behaviour	Three Factor Eating Questionnaire (TFEQ)	×	×	×	×		×	×
Sleeping	Epworth Sleepiness Scale (ESS), Pittsburgh Sleep Quality Index (PSQI)	×	×	×	×		×	×
Stress and mood	Perceived Stress Scale (PSS), Profile of Mood Scale (POMS)	×	×	×	×		×	×
Quality of life	WHO Quality of Life questionnaire	x			x		x	x
Work ability	Work Ability Index questionnaire	x			x		x	x
Cost-effectiveness	Questionnaire designed by the PREVIEW research group	x			x		x	x

^a^ In a subgroup (*n* = 250) in Helsinki and Auckland; ^b^ In a subgroup (*n* = 120) in Copenhagen, Maastricht, Navarra and Nottingham.

**Table 4:** Number and age distribution of participants recruited for the PREVIEW intervention trial.

Site	Pre-Screened	Screened	Randomised (*n*)	Men (*n*)	Women (*n*)	Age 25–45 Years (*n*)	Age 46–54 Years (*n*)	Age 55–70 Years (*n*)	Mean Age Years (SD)
**UCPH**	2061	908	379	159	220	86	62	233	54.2 (10.9)
**HEL**	1269	633	289	88	201	39	33	221	58.2 (8.9)
**UM**	675	553	203	94	109	42	17	145	56.6 (10.0)
**UNOTT**	3914	979	264	102	162	95	42	133	51.6 (12.0)
**UNAV**	1740	732	307	93	214	145	82	93	47.5 (10.6)
**MU**	1190	488	368	87	281	190	7	158	47.8 (12.0)
**UNSYD**	3108	595	195	56	139	59	36	102	53.0 (10.8)
**UOA**	1654	584	321	77	244	156	47	103	47.0 (11.4)
**Total**	15,611	5472	2326	756	1570	812	326	1188	51.6 (11.6)

Abbreviations for study sites: UCPH = University of Copenhagen (Denmark); HEL = University of Helsinki (Finland); UM = Maastricht University (The Netherlands); UNOTT = University of Nottingham (UK); UNAV = University of Navarra (Spain); MU = Medical University of Sofia (Bulgaria); UNSYD = University of Sydney (Australia); UOA = University of Auckland (New Zealand).

**Table 5:** Number of participants, age, anthropometric results, blood chemistry, and blood pressure for all intervention groups, assessed at baseline (CID1) before weight reduction. The results are shown as the mean (±SD).

	HP: Higher Protein (25 E%) Moderate Carbohydrate (45 E%) Low GI (≤50) Diet		MP: Moderate Protein (15 E%) Higher Carbohydrate (55 E%) Medium GI (≥56) Diet	
	Moderate-Intensity Physical Activity	High-Intensity Physical Activity	Moderate-Intensity Physical Activity	High-Intensity Physical Activity
No. (men/women)	556 (184/372)	556 (177/379)	559 (180/379)	553 (179/374)
Age, years	51.6 ± 11.5	51.8 ± 11.7	51.4 ± 11.2	51.4 ± 11.8
Anthropometrics				
Height, cm	168 ± 9	168 ± 9	168 ± 9	168 ± 10
Weight, kg	99.3 ± 20.8	100.6 ± 21.0	101.6 ± 22.6	98.7 ± 20.9
Body Mass Index, kg/m^2^	35.1 ± 6.5	35.6 ± 6.7	35.7 ± 6.6	35.0 ± 6.4
Waist circumference, cm	109.6 ± 15.2	111.0 ± 15.3	111.1 ± 15.4	109.6 ± 14.5
Hip circumference, cm	117.6 ± 14.5	118.8 ± 14.8	119.2 ± 13.9	117.8 ± 13.8
Body fat (% of weight)	43.0 ± 7.5	43.5 ± 7.5	43.5 ± 7.9	43.1 ± 7.8
Blood chemistry and blood pressure				
f P-glucose, mmol/L	6.2 ± 0.8	6.2 ± 0.6	6.2 ± 0.7	6.2 ± 0.8
2hP-glucose, mmol/L	7.8 ± 2.3	7.7 ± 2.25	7.5 ± 2.2	7.7 ± 2.1
fP-insulin, mU/L	13.6 ± 7.9	14.0 ± 8.7	13.2 ± 7.7	13.1 ± 7.2
HbA1c, mmol/mol	36.6 ± 3.9	36.8 ± 3.9	36.7 ± 4.2	36.7 ± 4.0
Cholesterol, mmol/L	5.2 ± 1.0	5.1 ± 1.0	5.3 ± 1.0	5.1 ± 1.0
LDL cholesterol, mmol/L	3.3 ± 0.9	3.2 ± 0.8	3.3 ± 0.9	3.2 ± 0.8
HDL cholesterol, mmol/L	1.3 ± 0.3	1.3 ± 0.3	1.3 ± 0.3	1.3 ± 0.3
Triglycerides, mmol/L	1.5 ± 0.8	1.5 ± 0.7	1.5 ± 0.9	1.5 ± 0.8
CRP, mg/L	5.3 ± 6.3	6.0 ± 8.7	5.2 ± 5.1	5.1 ± 7.5
Systolic BP, mmHg	128.5 ± 15.6	129.7 ± 16.2	128.7 ± 16.3	129.3 ± 15.5
Diastolic BP, mmHg	78.2 ± 10.9	77.6 ± 11.2	78.4 ± 11.5	78.3 ± 10.7

GI = glycaemic index; BP = blood pressure.

## Supplementary Materials

The following are available online at www.mdpi.com/2072-6643/9/6/632/s1, Table S1: Inclusion and exclusion criteria in the PREVIEW screening, Table S2: Questionnaires on moderators, mediators, behaviour, and social environment.
