# Supplementary material for: PREVIEW: Prevention of Diabetes through Lifestyle Intervention and Population Studies in Europe and around the World. Design, Methods, and Baseline Participant Description of an Adult Cohort Enrolled into a Three-Year Randomised Clinical Trial
Source: Nutrients. 2017 Jun 20;9(6):632. doi: 10.3390/nu9060632 (PMC5490611; doi:10.3390/nu9060632)
Supplement: Supplementary file 1 [file nutrients-09-00632-s001.zip › nutrients-199725-supplementary.pdf]

Supplementary Table S1: Inclusion and exclusion criteria in the PREVIEW screening.

Inclusion criteria:

1) Age: 25 to 70 years
2) Body mass index: ≥25.0 kg/m^2
3) Pre-diabetes, assessed immediately during screening by HemoCue™ (Angelholm, Sweden), Reflotron™ (Roche diagnostics, Switzerland) or EML105 Radiometer (Copenhagen, Denmark): The criteria from WHO/IDF (International Diabetes Foundation) for assessing pre-diabetes will be used as the formal inclusion criteria, i.e. having:

   - Impaired Fasting Glucose (IFG): Fasting venous plasma glucose concentration 5.6–6.9 mmol/l or
   - Impaired Glucose Tolerance (IGT): Venous plasma glucose concentration of 7.8–11.0 mmol/l at 2 h after oral administration of 75 g glucose (oral glucose tolerance test, OGTT), with fasting plasma glucose less than 7.0 mmol/l.
4) Signed informed consent.
5) Smoking is allowed, provided subjects have not recently (within one month) changed habits. However, smoking status is monitored throughout the study and used as a confounding variable.
6) Motivation and willingness to be randomized to any of the groups and to do his/hers best to follow the given protocol.
7) Able to participate at Clinical Investigation Days during normal working hours.

Exclusion criteria:

A. Medical conditions as known by the subjects:

1) Diabetes mellitus (other than gestational diabetes mellitus);

2) Significant cardiovascular disease including current angina; myocardial infarction or stroke within the past six months; heart failure; symptomatic peripheral vascular disease;

3) Systolic blood pressure above 160 mmHg and/or diastolic blood pressure above 100 mmHg whether on or off treatment for hypertension. If being treated, no change in drug treatment within the last three months. There will be no criteria for blood pressure limits in children and adolescents;

4) Advanced chronic renal impairment;

5) Significant liver disease, e.g., cirrhosis (fatty liver disease allowed);

6) Malignancy which is currently active or in remission for less than five years after last treatment (local basal and squamous cell skin cancer allowed);

7) Active inflammatory bowel disease, celiac disease, chronic pancreatitis, or other disorder potentially causing malabsorption;

8) Previous bariatric surgery;

9) Chronic respiratory, neurological, musculoskeletal or other disorders where, in the judgement of the investigator, participants would have unacceptable risk or difficulty in complying with the protocol (e.g. physical activity program);

10) A recent surgical procedure until after full convalescence (investigators judgement);

11) Transmissible blood-borne diseases, e.g., hepatitis B, HIV;

12) Psychiatric illness (e.g. major depression, bipolar disorder).

B. **Medication**:

13) Use, currently or within the previous three months, of prescription medication that has the potential of affecting body weight or glucose metabolism, such as glucocorticoids (but excluding inhaled and topical steroids; bronchodilators are allowed), psychoactive medication, epileptic medication, or weight loss medications (either prescription, over the counter or herbal). Low-dose antidepressants are allowed if they, in the judgement of the investigator, do not affect weight or participation to the study protocol. Levothyroxine for treatment of hypothyroidism is allowed if the participant has been on a stable dose for at least three months.

C. Personal/Other:

14) Engagement in competitive sports;

15) Self-reported weight change of >5 % (increase or decrease) within two months prior to screening;

16) Special diets (e.g. vegan, Atkins) within two months prior to study start. A lacto-vegetarian diet is allowed;

17) Severe food intolerance expected to interfere with the study;

18) Regularly drinking >21 alcoholic units/week (men), or >14 alcoholic units/week (women);

19) Use of drugs of abuse within the previous 12 months;

20) Blood donation or transfusion within the past one month before baseline or CIDs;

21) Self-reported eating disorders;

22) Pregnancy or lactation, including plans to become pregnant within the next 36 months.

23) No access to either phone or Internet (this is necessary when being contacted by the instructor's during the maintenance phase);

24) Inadequate understanding of national language;

25) Psychological or behavioural problems which, in the judgement of the investigator, would lead to difficulty in complying with the protocol.

Supplementary Table S2: Questionnaires on moderators, mediators, behaviour and social environment.

2a. General Description

Level	Variables (moderators, mediators, confounders)	Instrument	Items
Environmental variables	**Recommended physical activity availability and accessibility**	Influence on Physical activity Instrument [IPAI subscale environment; Donahue, Mielenz, Sloane, Callahan and Devellis, 2006]	5
	**Physical inactivity temptations**	Subscale competing demands from the Temptation to not exercise scale [Hausenblas et al. 2001]	6
	**Recommended food availability and accessibility**	Self-constructed items	7
	**Food temptations**	Self-constructed items	7
Social/ interpersonal variables	**Social support for diet and exercise**	Social support for diet and exercise scales [Sallis, Grossmann, Pinski, Patterson and Nader, 1987]	46
	**Teachable moments**	Social Readjustment Rating Scale [Holmes and Rahe, 1967]	8 + X (Filter)
Individual variables	**Socio-demographic and economic status**	European Social Survey and International Social Survey (choice of items)	18
	**Previous negative attempts of weight reduction**	Self-constructed items	2
	**Habit strength** of physical inactivity and poor diet	Habit strength measure [Wood, Tam and Guerro Witt, 2005]	12
	**Intention**	Self-constructed items [adapted from Schwarzer and Renner, 2011]	3
	**Self-efficacy** (physical activity and nutrition)	The nutrition self-efficacy scale and The physical exercise self-efficacy scale [Schwarzer and Renner, 2005]	5 + 5
	**Causal attributions** (for weight outcomes)	Attributional weight outcome scale [Brubacker, 1988]	15
	**Self-regulation goal adjustment**	Goal Adjustment Scale [Wrosch et al., 2003]	10
	**Coping** self-efficacy	Coping self-efficacy for physical activity and healthful nutrition [Schwarzer and Renner, 2000]	11+ 3

Level	Variables (moderators, mediators, confounders)	Instrument	Items
	**Perceived stress**	Perceived Stress Scale [PSS; Cohen, 1983]	10
	**Outcome expectancies**	Outcome expectancy of behaviour change (subscale for change of nutrition habits and subscale for exercise [Renner and Schwarzer, 2005]	12 + 13
	**Self-regulation of motivation**	Treatment self-regulation questionnaire for diet and exercise [TRSQ; Levesque et al., 2007]	15 + 15
Secondary endpoints	**Quality of Life**	WHOQOL-BREF [The WHOQOL Group, 1998]	26
	**Work ability** [a)]	Work Ability Index [WAI; Tuomi, et al., 1998]	24

a) employees

2b. Questionnaire List "Other Lifestyle Variables"—ADULTS

Variables (moderators, mediators, confounders)	Instrument (Reference)
**Recommended physical activity availability and accessibility**	Influence on Physical Activity Instrument [IPAI subscale environment; Donahue, Mielenz, Sloane, Callahan and Devellis, 2006]
**Physical inactivity temptations**	Subscale competing demands from the Temptation to not exercise scale [Hausenblas, Nigg, Dannecker, Symons Downs, Gardner, Fallon, Focht, Loving, 2001]
**Recommended food availability and accessibility**	Self-constructed items
**Food temptations**	Self-constructed items
**Social support for diet and exercise**	Social support for diet and exercise scales [Sallis, Grossmann, Pinski, Patterson and Nader, 1987]
**Teachable moments**	Social Readjustment Rating Scale [Miller and Rahe, 1997]
**Socio-demographic and economic status**	European Social Survey and International Social Survey (choice of items)
**Previous negative attempts of weight reduction**	Self-constructed items
**Habit strength** of physical inactivity and poor diet	Habit strength measure [Wood, Tam and Guerro Witt, 2005]
**Intention**	Self-constructed items [adapted from Schwarzer and Renner, 2010]
**Self-efficacy** (physical activity and nutrition)	The nutrition self-efficacy scale and The physical exercise self-efficacy scale [Schwarzer and Renner]
**Causal attributions** (for weight outcomes)	Attributional weight outcome scale [Brubaker, 1988]
**Self-regulation goal adjustment**	Goal Adjustment Scale [Wrosch, Scheier, Miller, Schulz, Carver, 2003]
**Coping** self-efficacy	Coping self-efficacy for physical activity and healthful nutrition [Schwarzer and Renner, 2000]
**Perceived stress**	Perceived Stress Scale [PSS; Cohen, 1983]
**Outcome expectancies**	Outcome expectancy of behaviour change (subscale for change of nutrition habits and subscale for exercise [Renner and Schwarzer, 2010]
**Self-regulation of motivation**	Treatment self-regulation questionnaire for diet and exercise [TRSQ; Levesque, Williams, Elliot, Pickering, Bodenhamer and Finley, 2007]

2c. Questionnaire List "Secondary Outcomes"—Adults

Questionnaire no	Variables (moderators, mediators, confounders)	Instrument (Reference)
**Q-25**	**Quality of Life**	WHOQOL-BREF [The WHOQOL Group, 1998]
**Q-26**	**Work ability** [a)]	Work Ability Index [WAI; Tuomi, et al., 1998]

References for Supplementary Table S2

Brubaker BH. An attributional analysis of weight outcomes. Nurs Res 1988;37(5):282-87.

Cohen S, Kamarck T, Mermelstein R. A global measure of perceived stress. J Health Soc Behav. 1983;24(4):385-96.

Donahue KE, Mielenz TJ, Sloane PD, Callahan, LF, Devellis, RF. Identifying supports and barriers to physical activity in patients at risk for Diabetes. Prev Chronic Dis [serial online] 2006;3:1-12. Internet: http://www.ncbi.nlm.nih.gov/pmc/articles/PMC1779283/pdf/PCD34A119.pdf [18.6.13].

Hausenblas HA, Nigg CR, Dannecker EA, Symons Downs D, Gardner RE, Fallon EA, et al. A missing piece of the transtheoretical model applied to exercise: Development and validation of the temptation to not exercise scale. Psychol Health 2001;16:381-90. http://dx.doi.org/10.1080/08870440108405514.

Levesque CS, Williams GC, Elliot D, Pickering MA, Bodenhamer B, Finley PJ. Validating the theoretical structure of the Treatment Self-Regulation Questionnaire (TSRQ) across three different health behaviors. Health Educ Res 2007;22(5):691-702.

Michie S, Abraham C, Whittington C et al. Effective Techniques in healthy eating and physical activity interventions: A meta-Regression. Health Psych 2009;28:690-701

Michie S, Richardson M, Johnston M et al. The behaviour change technique taxonomy (v1) of 93 hierarchically clustered techniques: building an international consensus for the reporting of behaviour change techniques. Annals Behav Medicine 2013;46:81-95

Miller MA, Rahe RH. Life changes scaling for the 1990s. J Psychosom Res 1997;43(3):279-92.

Greaves CJ, Sheppard KE, Abraham C et al. Systematic review of reviews of intervention components associated with increased effectiveness in dietary and physical activity interventions. BMC Public Health 2011;11:119

Olander EK, Fletcher H, Williams S et al. What are the most effective techniques in changing obese individuals' physical activity self-efficacy and behaviour: A systematic review and meta-analysis. Int J Behav Nutr Phys Act 2013;10:29

Prochaska JO, DiClemente CC. Stages of change in the modification of problem behaviors. In: Hersen M, Eisler RM, Miller P (eds) Progress on behavior modification. Sycamore Press, Sycamore, 1992; pp 184-214

Renner B, Schwarzer R. Risk and Health Behaviors. Documentation of the Scales of the Research Project: "Risk Appraisal Consequences in Korea" (RACK) 2nd ed. International University Bremen & Freie Universität Berlin, 2010.

Tuomi K, Ilmarinen J, Jahkola A, Katajarinne L, Tulkki A. Work Ability Index. 2nd revised edn. Helsinki: Finnish Institute of Occupational Health, 1998.

Sallis JF, Grossmann RM, Pinski RB, Patterson TL, Nader PR. The development of scales to measure social support for diet and exercise behaviors. Prev Med 1987;16:825-36.

Schwarzer R, Renner B. Social-cognitive predictors of health behavior: Action selfefficacy and coping self-efficacy. Health Psychol 2000;19: 487-95.

Schwarzer R, Renner B. Health-Specific Self-Efficacy Scales. Internet: http://userpage.fu-berlin.de/health/healself.pdf (accessed 20 June 2013)

WHOQOL group. WHOQOL-BREF: Introduction, administration, scoring and generic version of the assessment (field trial version). WHO Geneva, Switzerland, December 1996.

Wood W, Tam L, Guerro Witt M. Changing circumstances, disrupting habits. J Pers Soc Psychol 2005;88:918–33. DOI: 10.1037/0022-3514.88.6.918.

Wrosch C, Scheier MF, Miller GE, Schulz R, Carver CS. Adaptive self-regulation of unattainable goals: goal disengagement, goal reengagement, and subjective well-being. Pers Soc Psychol Bull 2003;29:1494-1508.
